# Combining ancillary soil data with VisNIR spectra to improve predictions of organic and inorganic carbon content of soils

**DOI:** 10.1016/j.mex.2018.05.019

**Published:** 2018-06-02

**Authors:** Patrick Filippi, Stephen R. Cattle, Thomas F.A. Bishop, Edward J. Jones, Budiman Minasny

**Affiliations:** The University of Sydney, School of Life and Environmental Sciences, Sydney Institute of Agriculture, Sydney, New South Wales, Australia

**Keywords:** Soil carbon measurement with visible near infrared spectroscopy, Visible near-infrared spectroscopy, Ancillary soil data, Organic carbon, Inorganic carbon, Carbonate, Cubist

## Abstract

While traditional laboratory methods of determining soil organic carbon (SOC) content are generally simple, this becomes more challenging when carbonates are present in the soil; such is commonly found in semi-arid areas. Additionally, soil inorganic carbon (SIC) content itself is difficult to determine. This study uses visible near infrared (VisNIR) spectra to predict SOC and SIC contents of samples, and the impact of including soil pH and soil total carbon (STC) data as predictor variables was evaluated. The results indicated that combining available soil pH and STC content data with VisNIR spectra dramatically improved prediction accuracy of the Cubist models. Using the full suite of predictor variables, Cubist models trained on the calibration dataset (75%) could predict the validation dataset (25%) for SOC content with a Lin’s concordance correlation coefficient (LCCC) of 0.94, and an LCCC of 0.83 for SIC content. This is compared to an LCCC of 0.81 and 0.35 for SOC and SIC content, respectively, when no ancillary soil data was included with VisNIR spectra as predictor variables. These results suggest that there may be promise for using other readily available soil data in combination with VisNIR spectra to improve the predictions of different soil properties.

• It can be laborious and expensive to measure soil organic and inorganic carbon content with traditional laboratory methods, and there has been recent focus on using spectroscopic techniques to overcome this.
• This study demonstrates that combining ancillary soil data (pH and total carbon content) with these spectroscopic techniques can considerably improve predictions of SOC and SIC content.

## Graphical abstract

**Figure:**
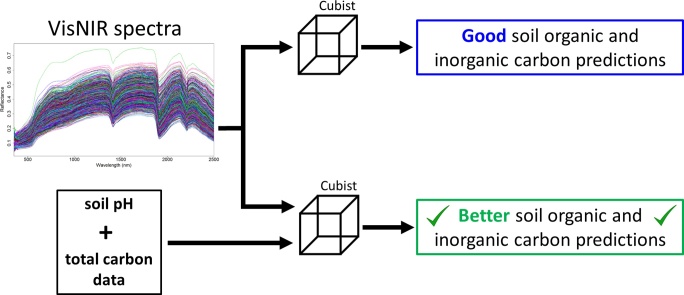

**Specifications Table**

**Table:** 

**Subject Area**	*Agricultural and Biological Sciences*
**More specific subject area:**	*Soil Science*
**Method name:**	*Soil carbon measurement with visible near infrared spectroscopy*
**Name and reference of original method**	McCarty, G.W., Reeves, J.B., Reeves, V.B., Follett, R.F. and Kimble, J.M., 2002. Mid-infrared and near-infrared diffuse reflectance spectroscopy for soil carbon measurement. *Soil Science Society of America Journal*, *66*(2), pp.640–646.
**Resource availability**	*NA*

## Method details

In soils that do not contain soil inorganic carbon (SIC), traditional laboratory methods of soil organic carbon (SOC) content determination are generally simple, and usually analogous to those for soil total carbon (STC) content determination. However, when carbonates are present in soil samples, such as in arid and semi-arid regions, the determination of SOC content becomes more challenging. As the sum of SOC and SIC content is equal to the STC content of a sample, at least two of the three components must be determined. However, due to the costly nature of measuring SOC and SIC content by traditional laboratory methods, this is often unfeasible.

The use of spectroscopic tools, such as visible near infrared (VisNIR), to predict the different components of soil carbon (SOC and SIC) has been extensively studied in recent years due to the cost and labour savings that come with these approaches [1]. Both SIC and SOC content are generally well predicted by these approaches [2], as there are specific VisNIR wavelengths that are heavily associated with these different components of soil carbon [3]. Most studies solely use VisNIR spectra as input variables for predicting SOC and SIC content [2], however, there are opportunities to combine VisNIR spectra with other useful and readily available soil information to further improve predictions. For example, soil pH data is commonly available as it can be easily determined by traditional laboratory methods, and including this information as a predictor variable could be advantageous due to the relationship that pH has with both SOC and SIC content. In this study, SOC and SIC content of samples in a semi-arid region in Australia are predicted using Cubist models, and the impact of combining VisNIR spectra data with soil pH and STC data as predictor variables is analysed.

## Materials and methods

### Study are and soil dataset

This study uses soil data collected from a semi-arid area surrounding the township of Hillston, in south-west NSW. The study area is ∼2500 km^2^ in size, and primarily consists of largely flat alluvial floodplains, with some rocky outcrops at higher elevation. The soils on the floodplain are mainly grey, brown and red Vertisols (IUSS Working Group WRB 2014), with sandier soils of largely aeolian origin at the higher points. Rainfall at Hillston is low, with a mean annual rainfall of 372 mm. The study area is subject to hot summers and cool winters, with a mean minimum temperature of 17.7 °C in summer and 4.5 °C in winter, and mean maximum temperatures of 31.2 °C in summer and 15.9 °C in winter [4].

Soil samples from 80 locations from a soil survey conducted in 2002 are used, as well as 140 soil cores from a soil survey from 2015 [5]. Samples were extracted from soils under a variety of land uses, including irrigated and dryland cropping, irrigated perennial horticulture, and rangeland grazing. Many of the same sites were sampled in both surveys (*n* = 70), as the locations were georeferenced. The subsampling intervals in the surveys differed, with the 2002 survey sampled at 0–0.2 and 0.3–0.4 m, and the 2015 sampled at 0–0.1, 0.1–0.3 and 0.3–0.5 m. In total, the soil dataset consists of 399 soil samples.

### Traditional laboratory methods

All of the soil samples were air-dried and then ground through a 2 mm sieve. Prior to laboratory analysis, all samples were tested for the presence of soil inorganic carbon (SIC). A ∼1 g subsample of ground soil was placed on a ceramic plate and a few drops of 1 M hydrochloric acid (HCl) were placed directly onto the sample. Any sample that showed an effervescence reaction was considered to contain calcium carbonate, the most prominent form of SIC in these soils. An additional subsample (∼10 g) was then taken and finely ground (<53 μm) using a Fritsch Mortar Grinder Pulverisette 2 (Fritsch, Germany) for 4 min at 50–60 Hz frequency. Soil total carbon (STC) content was determined by the combustion method with the Leco1 CHN analyser for 2002 samples, and the Elementar vario MAX CNS for 2015 samples. The Elementar vario MAX CNS and the Leco1 CHN analyser are very similar in their analytical approach, and both use the combustion technique. Soil organic carbon content for 2002 samples was determined by treating samples with 2 M HCl to remove inorganic carbon, and then analysing by the Leco1 CHN analyser [6]. For 2015 samples, SOC content was determined by the Walkely-Black method, which is a wet oxidation technique that uses chromic acid [7]. The Walkely-Black method was used, as it is one of the more rapid traditional laboratory methods of measuring SOC content. To estimate SIC content, the difference between STC and SOC contents was used. For 2002 samples, the SOC content was determined immediately, however, STC was determined from archived samples 13 years after sampling. While there may be potential drawbacks of analysing soil samples that have been archived for many years, most studies in the literature that have analysed the impact of archiving on soil carbon levels have found that this is negligible (e.g [8].).

### Spectral predictions

#### VisNIR spectral acquisition and processing

Archived soil samples from both the 2002 and 2015 soil surveys (*n* = 399) were scanned by visible near infrared (VisNIR) with an Agrispec portable spectrophotometer with a contact probe attachment (Analytical Spectral Devices, Boulder, Colorado) on the dried and ground soil samples. To reduce signal-to-noise ratios of the spectra, three scans of each sample were performed, from which an averaged reflectance spectrum was derived. Calibration of the instrument was made with a Spectralon white tile and was re-calibrated after every 15 scans, or five samples.

Pre-processing of the VisNIR spectra was performed, which included splicing the discontinuities at VisNIR detector junctions (1000 and 1800 nm), and then converting reflectance to absorbance. Smoothing of the spectra was then performed using a Savitzky-Golay filter (Savitzky and Golay, 1964), and wavelengths of VisNIR outside the 500–2450 nm were removed, and the remaining wavelengths were resampled at 10 nm intervals to reduce data quantity. A Standard Normal Variate (SNV) baseline correction (Barnes et al. 1989) was then performed on the remaining spectra.

#### Prediction models

Along with the VisNIR spectra, the mid-depth of the sample was included as a predictor variable in the models, to ensure that the depth was taken into account when predicting. In addition to these, soil pH and STC content (measured by traditional laboratory approaches described in the methodology above) were included as predictor variables, as this data was available for all samples. For each soil property (SOC and SIC), five variations of model inputs was tested. These included VisNIR and mid-depth (model A); VisNIR, mid-depth and pH (model B); VisNIR, mid-depth and STC content (model C); VisNIR, mid-depth, pH and STC content (model D); and finally mid-depth, pH and STC content without VisNIR spectra (model E).

Cubist models were used to predict SOC and SIC content, with each of the five combinations of predictor variables. Cubist is a regression rule technique that essentially functions by creating one or more rules, where each rule is a linear model of the predictor variables [9]. To test prediction quality, 75% of the dataset was used as calibration, and the remaining 25% was used as validation. These datasets were selected by performing a Latin hypercube sampling of the VisNIR spectra, pH, STC, mid-depth, and the response variables to ensure that both the validation and calibration datasets were appropriately represented. The maximum number of rules used in the Cubist models was 10. The bagging, or bootstrap aggregating, method was used to generate different models from varying realisations of the calibration dataset with the aim to enhance the prediction and also estimate uncertianty of the model [18]. This approach uses repeated random sampling, where the calibration dataset of size *N* is replaced to calculate the *B* bootstrap. Each bootstrap has the same size as the calibration dataset, but does not contain the same samples. In total, there were 50 bootstraps, meaning that 50 Cubist models were generated for each soil property and each combination of predictor variables. The mean was then calculated from the 50 soil property predictions for each sample in the calibration dataset. The statistics used to test the model quality included Lin’s concordance correlation coefficient (LCCC), root mean square error (RMSE), bias (mean of the residuals), and *R*^2^, with this being tested on both the calibration and validation datasets. All statistical analyses were performed in the statistical program R [10].

## Results

### Summary statistics of laboratory-measured soil properties

Overall, SOC contents of the soil samples are low, with a mean value of 0.58% and values ranging from 0.09 to 1.77% (Table 1). The SIC contents were quite variable, with a minimum of 0, a maximum of 1.60%, and a mean of 0.04%. The STC content of samples ranged from 0.63 to 1.85%, and possessed a mean of 0.63%. The mean pH of all samples was slightly alkaline at 7.57, but ranged considerably from 5.02 to 9.62 (Table 1).

**Table 1:** Summary statistics of laboratory-measured soil organic carbon (SOC), soil inorganic carbon (SIC), and soil total carbon (STC) content (%), and soil pH of all samples at all sampling depths and all time points.

	SOC %	SIC %	STC%	pH
Mean	0.60	0.04	0.63	7.57
Median	0.58	0	0.60	7.49
Minimum	0.09	0	0.09	5.02
Maximum	1.77	1.60	1.85	9.62
Standard deviation	0.279	0.139	0.289	0.935
*n*	399	399	399	399

### Visible near infrared (VisNIR) spectroscopy predictions

**Fig. 1:**
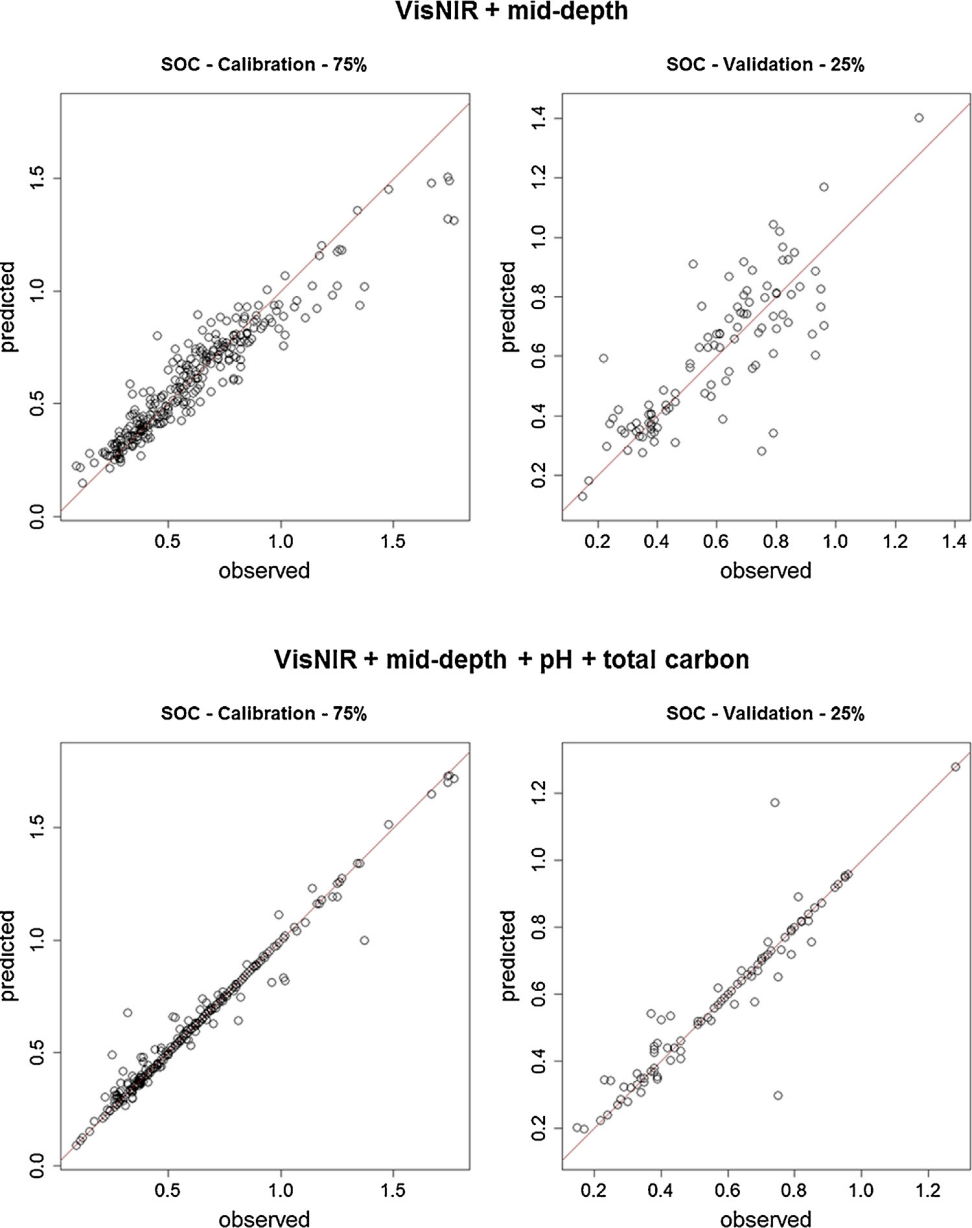
Observed vs. predicted soil organic carbon (SOC) content (%) values of calibration (left) and validation (right) datasets predicted with VisNIR spectra and mid-depth (top); and VisNIR spectra, mid-depth, soil pH and soil total carbon contents (bottom) as predictor variables in Cubist models.

**Fig. 2:**
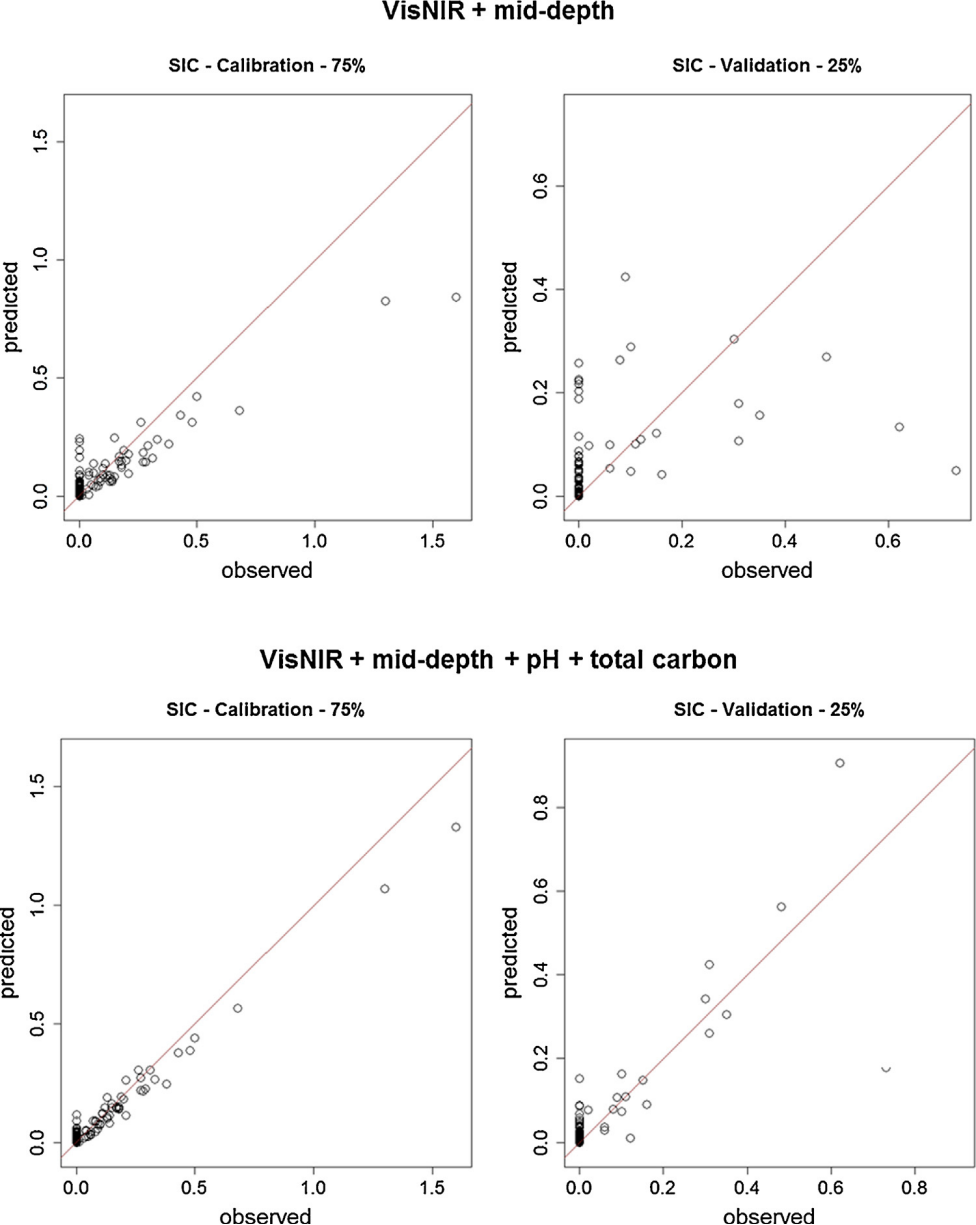
Observed vs. predicted soil inorganic carbon (SIC) content (%) values of calibration (left) and validation (right) datasets predicted with VisNIR spectra and mid-depth (top); and VisNIR spectra, mid-depth, soil pH and soil total carbon contents (bottom) as predictor variables in Cubist models.

Both SOC and SIC content of samples displayed a mild to strong relationship (Pearson’s correlation) with the predictor variables of STC content and soil pH (Table 2). The independently-validated statistics also showed that both SOC and SIC content of samples could be predicted with high accuracy using spectroscopic techniques (Fig. 1, Fig. 2; Table 3). Overall, SOC content was predicted with greater accuracy than SIC content, and the different combinations of model inputs had a clear impact on the prediction quality for both SOC and SIC content. The LCCC was primarily used for the assessment of model quality, as it is the fit of the 1:1 line of the observed and predicted values. It is also unit less, which makes it useful for comparing different models of the same soil property, as well as comparing models for different soil properties.

**Table 2:** Pearson’s correlation (*r*) between response variables (SOC and SIC content) and predictor variables (STC content and pH).

	SOC	SIC	STC	pH
SOC	1	–	–	–
SIC	−0.15	1	–	–
STC	0.86	0.35	1	–
pH	−0.36	0.39	−0.18	1

**Table 3:** Model quality statistics for SOC and SIC content predictions from Cubist models with varying combinations of input variables.

Attribute	Model	Input variables	Dataset	LCCC	R^2^	RMSE (%)	bias
Soil Organic Carbon (SOC)	A	VisNIR + mid-depth	Calibration	0.93	0.90	0.10	0.00
			Validation	0.81	0.67	0.14	0.01
	B	VisNIR + mid-depth + pH	Calibration	0.93	0.89	0.10	0.00
			Validation	0.81	0.67	0.14	0.01
	C	VisNIR + mid-depth + STC	Calibration	0.97	0.96	0.06	0.00
			Validation	0.90	0.84	0.09	0.02
	D	VisNIR + mid-depth + pH + STC	Calibration	0.98	0.98	0.05	0.00
			Validation	0.94	0.89	0.07	0.00
	E	mid-depth + pH + STC	Calibration	0.98	0.98	0.04	0.00
			Validation	0.92	0.86	0.11	0.01
Soil Inorganic Carbon (SIC)	A	VisNIR + mid-depth	Calibration	0.83	0.85	0.07	0.00
			Validation	0.35	0.14	0.12	0.02
	B	VisNIR + mid-depth + pH	Calibration	0.86	0.87	0.06	0.00
			Validation	0.52	0.29	0.11	0.01
	C	VisNIR + mid-depth + STC	Calibration	0.94	0.97	0.04	0.00
			Validation	0.82	0.73	0.06	0.01
	D	VisNIR + mid-depth + pH + STC	Calibration	0.97	0.98	0.03	0.00
			Validation	0.83	0.70	0.07	0.01
	E	mid-depth + pH + STC	Calibration	0.95	0.95	0.04	−0.00
			Validation	0.78	0.66	0.06	−0.00

For SOC content, it was clear that the inclusion of additional soil property data improved prediction results considerably, improving the predictions on the validation dataset from an LCCC of 0.81 for the model without ancillary soil data (model A), to an LCCC of 0.94 for the model that included all predictor variables (model D) (Table 3; Fig. 1). It can be seen in Fig. 1 that model D predicted SOC to ∼100% accuracy for multiple soil samples (where the points lie exactly on the 1:1 line) in both the calibration and validation plot, whereas this did not occur for model A. For SOC content, the inclusion of pH alone with VisNIR and mid-depth (model B) did not improve predictions, but the inclusion of STC content with VisNIR and mid-depth (model C) improved predictions significantly. Organic carbon content had a particularly high positive correlation (*r*) with total carbon content (0.86) and a weaker negative correlation with pH (−0.36), which explains their relative importance in the prediction of SOC content (Table 2). Overall, the inclusion of both pH and STC content together with VisNIR and mid-depth (model D) resulted in the best model. Interestingly, the second best model for predicting SOC content was with the model that contained no VisNIR spectra and only ancillary soil information as predictor variables (model E), with an LCCC of 0.92 on the validation dataset (Table 3). This indicates the high value of the ancillary soil information in predicting SOC content.

While SOC content could be predicted to higher accuracy, the inclusion of STC content and pH data as predictor variables with VisNIR and mid-depth was even more effective in improving SIC content predictions with Cubist models (Table 3; Fig. 2). For SIC content, VisNIR and mid-depth only models (model A) predicted the validation dataset very poorly, with an LCCC of 0.35, whereas the model with the full suite of input variables (model D) predicted the validation dataset to an accuracy of 0.83 LCCC (Table 3). While the inclusion of pH alone (model B) did not improve SOC content predictions, it made a noteworthy improvement from the simplest model (model A) in SIC content predictions to predict the validation dataset to an accuracy of 0.52 LCCC (Table 3). There was a slightly higher absolute correlation (*r*) with pH and SIC content (0.39) than there was with pH and SOC content (-0.36), although the correlation with STC and SIC contents was much weaker (0.35) than with STC and SOC contents (0.86) (Table 2). Overall, the Cubist models that included VisNIR spectra, mid-depth, pH and STC content as predictor variables proved to be the most accurate at predicting SIC content. Similarly to SOC content predictions, the model with mid-depth, soil pH and STC data and without VisNIR spectra was the second best model for predicting SIC content, with and LCCC of 0.78 when predicting on the validation dataset (Table 3).

Table 4 shows the five most important predictor variables for each Cubist model, giving the percentage of times where the variable was used in a condition, and the percentage of times it was used in a linear model. It was clear that the ancillary soil data played a significant role in both the conditions, and the models (Table 4). For example, in the model that contained the full-suite of predictors (model D), pH and STC were the most important variables for SOC content, and in the top three for SIC content (Table 4). In terms of important wavelengths for the different SOC models; 1400, 1900, and 2140 nm were important in both model A and B, and 570 nm was important in models A and C. For the SIC models, wavelength 570 nm appeared in both models A and C, 1480–90 nm in B and D. Higher wavelengths at 2220 nm for model A and 2270 nm for model C were also important predictors for SIC content (Table 4).

**Table 4:** Important predictor variables for each Cubist model, showing the attribute usage (%) for conditions and the models.

	Soil organic carbon			Soil inorganic carbon		
	Important Variable	Conditions (%)	Model (%)	Important Variable	Conditions (%)	Model (%)
**Model A**	**Depth**	97	32	**570 nm**	62	65
	**1900 nm**	47	94	**Depth**	47	12
	**1400 nm**	47	52	**2220 nm**	44	66
	**2140 nm**	43	50	**720 nm**	32	66
	**570 nm**	43	94	**690 nm**	10	66
**Model B**	**Depth**	97	49	**pH**	83	26
	**1400 nm**	47	100	**1170 nm**	51	25
	**1900 nm**	47	98	**810 nm**	29	23
	**2140 nm**	43	97	**1480 nm**	20	30
	**1890 nm**	32	98	**1390 nm**	10	9
**Model C**	**STC**	76	100	**570 nm**	86	84
	**570 nm**	58	37	**Depth**	51	47
	**Depth**	52	37	**STC**	35	63
	**2110 nm**	22	27	**2270 nm**	29	–
	**610 nm**	18	38	**1080 nm**	28	99
**Model D**	**pH**	97	50	**pH**	100	20
	**STC**	48	100	**1490 nm**	46	27
	**1380 nm**	10	52	**STC**	24	28
	**1760 nm**	4	46	**790 nm**	9	28
	**660 nm**	3	1	**1540 nm**	9	27
**Model E**	**pH**	100	46	**pH**	100	61
	**STC**	53	98	**STC**	17	61
	**Depth**	24	29	**Depth**	–	63
	–	–	–	–	–	–
	–	–	–	–	–	–

## Discussion

### Soil property predictions and predictor variable importance

Overall, both SOC and SIC contents of samples from the semi-arid region of Hillston could be accurately predicted with spectroscopic techniques. It was clear from the results that combining soil pH and STC content data as predictor variables with VisNIR spectra substantially improved the accuracy of both SOC and SIC content predictions compared to solely using VisNIR spectra.

In particular, SOC content was predicted with very high accuracy by the model that included VisNIR, mid-depth, pH and STC (model D), with an LCCC of 0.94 when predicted on the validation dataset compared to an LCCC of 0.81 for the model that contained only VisNIR and mid-depth (model A). This is logical, as SOC content is highly positively correlated (*r*) with STC content (0.86), and mildly negatively correlated with pH (−0.36). The importance of these ancillary data in predictions of SOC content was demonstrated in the model that only contained mid-depth, pH and STC (model E), where despite no VisNIR spectra being included in the model, the calibration dataset could still predict the validation dataset to an accuracy of 0.92 LCCC (Table 3). When predicting on both the calibration and validation datasets with model D, it was apparent that SOC content was predicted with ∼100% accuracy for several samples, as can be seen in Fig. 1. These very accurate predictions can be logically explained. It is likely that the Cubist model is detecting that there is no inorganic carbon in the sample, and because Cubist models are essentially rule-based decision trees, the model is simply assigning the inputted STC value as the SOC content prediction. There are particular VisNIR wavelengths that are associated with SIC [11], and if these wavelengths of the scanned sample do not possess the appropriate reflectance, the model is likely determining that there is no SIC present in the sample. While the inclusion of soil pH alone with VisNIR and mid-depth did not improve SOC predictions (model B), when this was included in combination with STC (model D), the predictions were slightly better than model C (VisNIR, mid-depth and STC), suggesting that there is an advantageous interaction occurring with STC and pH in these models.

Studies have reported that the accuracy of predicting SOC and SIC content of samples with VisNIR is generally quite similar [12,13], although this depends on a number of factors. In our study, this was not the case, and the best combination of covariates (model D) predicted the validation dataset with an LCCC of 0.94 for SOC content, and 0.83 for SIC content (Table 3). Again the value of ancillary soil data was exemplified, with the model that contained mid-depth, pH and STC, and no VisNIR spectra (model E) showing relatively high predictions of SIC content, with an LCCC of 0.78 on the validation dataset. A possible reason for the poorer predictions of SIC content compared to SOC content is due to the nature and distribution of the SIC dataset. The SIC dataset in our study is zero-inflated (contains many zero values), and consequently there are fewer samples that contain some amount of SIC in the training dataset. In addition, SIC content of samples was not directly measured by laboratory methods, and was determined by the difference between measured SOC and STC content, which includes a greater amount of error. While there were multiple occurrences of SOC content being predicted to ∼100% accuracy, this was not the case for SIC content. This is logical, as all soil samples in the study contain some amount of SOC, even if it is a very small amount, but not all samples contain SIC. As a result, the model could not simply assign the SIC value as the STC value.

While the inclusion of soil pH as a predictor variable with VisNIR and mid-depth (model B) did not improve the SOC content predictions, it made a significant improvement in SIC content predictions. Soil pH was found to be positively correlated (*r*) with SIC content (0.39) and it is known that very alkaline pH levels indicate the presence of considerable amounts of carbonate in a soil. Soils that possess a pH of less than 7 (1:5 H_2_O) also commonly do not contain SIC (Wang et al. 2015). Although the correlation (*r*) of SIC with STC content was relatively weak (0.35), including STC content as a predictor improved predictions on the validation dataset from an LCCC of 0.35 for model A to 0.82 for model C. While combining pH with spectra improved SIC content predictions, this positive impact seemed to be masked when both pH and STC content were included as predictor variables together, with the LCCC of predictions on the validation dataset for model D only slightly better at 0.83.

The analysis of variable importance of the different models of SOC and SIC content showed that the ancillary soil data played a significant role in both the conditions and the models (Table 4). As expected, the most important wavelengths of the VisNIR spectra for the different models for both SOC and SIC content varied, however, there were a few wavelengths that were important for several models. In particular, 570 nm was in the top five most important predictors in model A and B for both SOC content and SIC content. Other studies have reported similar results, such as Viscarra Rossel et al. [2], where 570 nm was identified as an important predictor for SOC content, and Ostovari et al. [14], where 571 nm was an important predictor for calcium carbonate (CaCO_3_). For SIC content, higher wavelengths at 2220 nm for model A and 2270 nm for model C were identified as important predictors, which is also commonly reported by other similar studies (e.g [11,14,15].).

### Limitations and opportunities

It must be acknowledged that including STC content data with VisNIR spectra to predict the SOC content of a sample is likely impractical and unnecessary for many studies. The prediction of SOC content with spectra alone was of high quality in our study, and this has been the case for many other studies [2]. Our study, however, demonstrates that there is considerable benefit in measuring STC content and including this in model predictions with VisNIR to predict SIC content. Although inorganic carbon is not found in all soils, this approach could be particularly appropriate for areas that typically possess soils with carbonates, such as arid and semi-arid areas. Our results also suggest that there is considerable benefit in including soil pH data with VisNIR spectra to predict SIC content. Soil pH data is also typically more available than STC content data, as it can be rapidly and cheaply measured by traditional laboratory methods. As soil pH is often correlated with different soil attributes such as nutrient availability, this also shows promise for combining soil pH data with spectra to predict other soil properties.

This also opens the discussion as to the possible benefits of combining other cheaply-measured and readily available soil data with VisNIR spectra to predict different soil properties, as there are many soil properties that are highly correlated with each other. For example, soil electrical conductivity (EC) is easily measured by traditional laboratory methods and hence this data is often available. It is known that EC is well correlated with other soil properties that are typically laborious to measure, such as soil particle size, and cation exchange capacity [16]. While studies often use ancillary soil data combined with pedotransfer functions to estimate the value of a soil property [17], there are no studies, to our knowledge, that use ancillary soil data in combination with spectra to predict another soil property. There are some limitations to adopting this approach, as including additional soil property data with spectra in predictive models requires that both the training dataset and prediction dataset possess a value for that soil property. Despite this, when ancillary soil data is available, it could be very useful in improving the quality of soil spectroscopic predictions.

## Conclusions and future directions

It was clear from the results in this study that the inclusions of soil pH and STC content as predictor variables substantially improved the prediction of both SOC and SIC content when combined with VisNIR wavelengths of the scanned soil samples. When combined with VisNIR spectra, soil pH data markedly improved the prediction of SIC content, which is a particularly significant finding as SIC content is difficult to measure by traditional laboratory techniques, whereas soil pH information is often readily available. The overall results from this study suggest that there is promise for including other readily available soil data with VisNIR to predict different soil properties, particularly when the soil property used as a predictor is correlated with the soil property to be predicted.

## References

[1] Miklos M., Short M.G., McBratney A.B., Minasny B. (2010). Mapping and comparing the distribution of soil carbon under cropping and grazing management practices in Narrabri, north-west New South Wales. Soil Res..

[2] Viscarra Rossel R.A., Walvoort D.J.J., McB ratney A.B., Janik L.J., Skjemstad J.O. (2006). Visible, near inf rared, mid inf rared or combined diffuse reflectance spectroscopy for simultaneous assessment of various soil properties. Geoderma.

[3] Cécillon L., Barthès B.G., Gomez C., Ertlen D., Genot V., Hedde M., Stevens A., Brun J.J. (2009). Assessment and monitoring of soil quality using near-infrared reflectance spectroscopy (NIRS). Eur. J. Soil Sci..

[4] Bureau of Meteorology – BOM (2017). Monthly Rainfall – Hillston Airport. http://www.bom.gov.au/climate/averages/tables/cw_075032_All.shtml.

[5] Filippi P., Cattle S.R., Bishop T.F.A., Odeh I.O.A., Pringle M.J. (2018). Digital soil monitoring of top- and sub-soil pH with bivariate linear mixed models. Geoderma.

[6] Tiessen H., Moir J.O., Carter M.R. (1993). Soil chemical analyses: total organic carbon. Soil Sampling and Methods of Analysis.

[7] Walkley A., Black I.A. (1934). An examination of the Degtjareff method for determining soil organic matter, and a proposed modification of the chromic acid titration method. Soil Sci..

[8] Lawrence C.R., Harden J.W., Xu X., Schulz M.S., Trumbore S.E. (2015). Long-term controls on soil organic carbon with depth and time: a case study from the Cowlitz River Chronosequence, WA USA. Geoderma.

[9] Quinlan J.R. (1993). Combining instance-based and model-based learning. Proceedings of the Tenth International Conference on Machine Learning.

[10] R Development Core Team (2017). R: A Language and Environment for Statistical Computing.

[11] Gaffey S.J. (1986). Spectral reflectance of carbonate minerals in the visible and near infrared (0.35–2.55 microns). Part 1: calcite, aragonite, and dolomite. Am. Mineral..

[12] Chang C.-W., Laird D.A., Mausbach M.J., Hurburgh C.R. (2001). Near-infrared reflectance spectroscopy–principal components regression analyses of soil properties. Soil Sci. Soc. Am. J..

[13] McCarty G., Reeves J., Reeves V., Follett R., Kimble J. (2002). Mid-infrared and near-infrared diffuse reflectance spectroscopy for soil carbon measurement. Soil Sci. Soc. Am. J..

[14] Ostovari Y., Ghorbani-Dashtaki S., Bahrami H.A., Abbasi M., Dematte J.A.M., Arthur E., Panagos P. (2018). Towards prediction of soil erodibility, SOM and CaCO3 using laboratory vis-NIR spectra: a case study in a semi-arid region of Iran. Geoderma.

[15] Mulder V.L., Plötze M., De Bruin S., Schaepman M.E., Mavris C., Kokaly R.F., Egli M. (2013). Quantifying mineral abundances of complex mixtures by coupling spectral deconvolution of SWIR spectra (2.1–2.4 μm) and regression tree analysis. Geoderma.

[16] Grisso R.D., Alley M.M., Holshouser D.L., Thomason W.E. (2005). Precision Farming Tools. Soil Electrical Conductivity.

[17] McBratney A.B., Minasny B., Cattle S.R., Vervoort R.W. (2002). From pedotransfer functions to soil inference systems. Geoderma.

[18] Breiman L. (1996). Bagging predictors. Mach. Learn..

